# A Feature Fusion Method with Guided Training for Classification Tasks

**DOI:** 10.1155/2021/6647220

**Published:** 2021-04-14

**Authors:** Taohong Zhang, Suli Fan, Junnan Hu, Xuxu Guo, Qianqian Li, Ying Zhang, Aziguli Wulamu

**Affiliations:** ^1^Department of Computer, School of Computer and Communication Engineering, University of Science and Technology Beijing (USTB), Beijing 100083, China; ^2^Beijing Key Laboratory of Knowledge Engineering for Materials Science, Beijing 100083, China; ^3^QingGong College, North China University of Science and Technology, Tangshan, Hebei 064000, China

## Abstract

In this paper, a feature fusion method with guiding training (FGT-Net) is constructed to fuse image data and numerical data for some specific recognition tasks which cannot be classified accurately only according to images. The proposed structure is divided into the shared weight network part, the feature fused layer part, and the classification layer part. First, the guided training method is proposed to optimize the training process, the representative images and training images are input into the shared weight network to learn the ability that extracts the image features better, and then the image features and numerical features are fused together in the feature fused layer to input into the classification layer for the classification task. Experiments are carried out to verify the effectiveness of the proposed model. Loss is calculated by the output of both the shared weight network and classification layer. The results of experiments show that the proposed FGT-Net achieves the accuracy of 87.8%, which is 15% higher than the CNN model of ShuffleNetv2 (which can process image data only) and 9.8% higher than the DNN method (which processes structured data only).

## 1. Introduction

In order to identify objects directly from images, researchers have proposed convolutional neural network (CNN), a deep learning model or multilayer perceptron which is like artificial neural networks, to regard each pixel of the image as a feature. CNN is commonly used to analyze visual images. The first generation of CNN is LeNet [1], proposed by LeCun in 1998. This network structure is proposed to solve the visual task of handwritten digit recognition, and it is one of the most representative structures in early CNNs. Since then, the most basic architecture of CNNs has been determined: the convolutional layer, the pooling layer, and the fully connected layer. In 2012, Alex Krizhevsky proposed the AlexNet [2] network structure, which proposed new activation function (ReLU), local response normalization (LRN), dropout, and data augmentation methods to improve the generalization ability of the network. AlexNet won the first place in the ILSVRC2012 [3], and CNNs have received extensive attention from researchers since then. After AlexNet, many excellent CNN models have appeared, and there are three main development directions: (a) deeper: the network layer is deeper, and the representative network is VggNet [4], ResNet [5]; (b) modularization: a modular network structure (Inception), the representative network is GoogleNet [6], Inceptionv2 [7], Inceptionv3 [8], and Inceptionv4 [9]; (c) faster: lightweight network model, for mobile devices, representative networks are SqueezeNet [10], MobileNet [11], ShuffleNet [12], MobileNetv2 [13], ShuffleNetv2 [14], and MobileNetv3 [15].

Images can provide feature information such as texture, morphology, and color for CNNs. When extracting features from images, images are always affected by various uncertain factors [16–18]. In order to reduce the impact of uncertainty, researchers use some data enhancement methods [19–21]. However, only image data is not adequate for some specific recognition tasks. For example, when a patient is diagnosed whether having a lung disease, the patient's x-ray film information and the other clinical symptoms would be applied together to consider the patient's condition and further propose a treatment plan. Here, x-ray film information is image data; the other clinical symptoms can be organized into structured data; the patient's condition or diagnosis is the prediction result of classifier. In this case, image data is not the unique and absolute criterion; the diagnosis should be made by the combination of image data and structured data.

**Figure 1:**
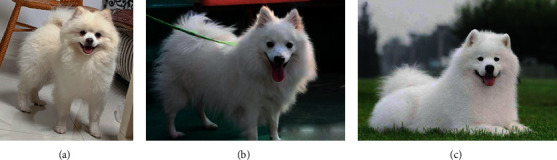
The image examples of Pomeranian (a), Japanese Spitz (b), and Samoyed (c).

Another example is about the recognition of three breeds of dogs, Pomeranian, Samoyed, and Japanese Spitz. If they are recognized only by images, as shown in Figure 1, it is difficult to have a high recognition rate because they have similar textures, appearances, and colors in the images. But the real sizes of the three dogs are different. The actual physical sizes could not be objectively reflected by different images. Because it is hard for the shooting distances to be the same in different images which are shot by different photographers and in different places. This kind of recognition problems should be executed by images and other information together to obtain a high recognition accuracy.

Therefore, in this paper, we design a novel framework to fuse image features (which are obtained by CNN method) with numerical features (which are obtained from structured data) together to solve this kind of classification problems. There are no such methods at present to combine CNN network with structured data in the same framework. Influenced by the idea of adaptive parameter selection in [22], the shared weight network is adopted as the training part designed by guided training. The fused features become a feature vector, which is input to the classifier. It should be noted that our approach is effective for the problems which should be solved comprehensively by image data and structured data together.

The contributions of this article are as follows:

1. A fusion framework FGT-Net is proposed, which has the capability of fusing image data and numerical data to enhance the representativeness of features for the further classification.
2. A guided training method is proposed. The training method can promote the framework to learn the features of images, so that the features of images belonging to the same class are as the same as possible.
3. The function of CNN structure is extended to structured data except for image data. It increases the ability of CNN to process image data and structured data at the same time and solves some specific problems which cannot be accurately classified according to images only.

There are many acronyms in this paper. The full names of all acronyms in this paper are listed in Table 1.

## 2. Related Work

### 2.1. Intraclass and Interclass Variance

At present, the main idea of classification of these categories with small gap is to reduce the intraclass variance and increase the interclass variance. There are a lot of researches on reducing the intraclass variance and increasing the interclass variance in the field of face recognition. When the traditional Softmax is used for training, the posterior probability of the sample's feature vector *x* (the input vector of the last fully connected layer) belongs to class *i* is *e*^*w*_*i*_*∗x*+*b*_*i*_^/∑_*j*=1_^*n*^*e*^*w*_*j*_*∗x*+*b*_*j*_^, where *n* is the number of classes, and **w** is weight of the last fully connected layer, **b** is bias. In [23–25], it is proposed to set *b*_*i*_ to 0, so *w*_*j*_*∗x*=||*w*_*j*_||*∗*||*x*||*∗*cos(*θ*_*j*_); *θ*_*j*_ represents the angle between *x* and the weight vector *w*_*j*_. In order to reduce the intraclass variance and increase the interclass variance, the authors in [26] proposed L-Softmax by adding angle constraint cos(*mθ*). On the basis of [26], SphereFace [23] normalized the module length of weight vector to 1. In order to further optimize the recognition effect, CosFace [24] and ArcFace [25] further normalized the module length of feature vector *x* to 1, and further proposed margin term cos(*θ*) − *m* in [24] and margin term cos(*θ*+*m*) in [25]. In addition, some researchers have proposed auxiliary loss function based on the existing loss function, such as Ringloss proposed by [27] and Qrthogonal loss function proposed by [28]. However, first of all, the same kind of images in face recognition comes from the same person, and the similarity is very large, while the images in our dataset belong to the same category from different dogs, Therefore, the original dataset of face recognition has reduced the intraclass variance to a certain extent, while our dataset has larger intraclass variance, which makes classification more difficult. In addition, these face recognition researches, whether face verification or face identify, in the actual recognition, either input two pictures for comparison to determine whether they are the same identity (face verification), or input an image, and compare with the existing image database, and determine whether the image belongs to the same category (face identify). In other words, face recognition needs an image database corresponding to the image to be recognized. The purpose of our research is to input only one sample and output the corresponding category of the sample directly; that is, we do not need to compare the sample database, so our task of identification is more difficult.

### 2.2. Multisize Detecting

Recently, there are many methods for detecting multi size targets. Singh and Davis [29] proposed scaling an image at different scales, extracting features at each scale, and fusing all features. The study in [30] detected the feature map of different resolutions, combined the prediction of multiple feature maps, and processed targets of various sizes. Cai et al. [31] used features of different resolutions to detect targets of different scales. The study in [32] combined bottom-up and top-down features to detect targets with different scales on different levels of feature maps. We find that these methods can only detect objects of different sizes in the same image. If two objects come from two images, these methods cannot distinguish the size of the two objects, because the scenes taken in these two pictures may be different. One may be obtained from a distance from the camera, and the other may be obtained from a relatively close distance. The paper [33] proposed a dynamic differential entropy (DDE) algorithm to extract the features of electroencephalogram signals. After that, the extracted DDE features were classified by convolutional neural networks. Therefore, here, we propose using auxiliary information to help further classification, such as weight and age, by fusing the features to distinguish objects in different images. To judge the three classes of dogs, only image data are not adequate. The supplement data, for example, real size or weight, need to be fused.

### 2.3. Feature Fusion

There have been some advances in the direction of feature fusion. The study in [34] fuses textual data and structured numerical data to improve the recognition effect, and this feature fusion method improves the accuracy of heart disease diagnosis. Yu et al. [35] proposed a generic data fusion model called attribute heterogeneous network fusion (AHNF), which encodes various internal relations between objects and fuses information from multiple data sources. Wang et al. [36] proposed a deep feature fusion method based on adaptive discriminant metric learning (DFF-ADML) to fuse different deep feature vectors of the same image. Cai et al. [37] constructed global feature vectors by fusing different images of the same object to achieve feature fusion. Tabik et al. [38] and Pan et al. [39] achieved feature fusion by fusing feature vectors obtained from multiple classification network models, and improved classification accuracy by combining the classification ability of multiple classifiers. Lai et al. [40] controlled the traffic lights by fusing the signals of traffic lights on different roads, to improve the congestion of the whole road network. Bin et al. [41] proposed using two deep neural networks to extract the features of urban structured numerical data and housing property structured data, respectively, and then fuse the two type features to achieve more accurate property value assessment for the real estate industry. Ma et al. [42] proposed an unsupervised framework based on generative adversarial network (GAN) [43] to realize the fusion of panchromatic images and low-resolution multispectral images, to obtain high-resolution multispectral images. Shao et al. [44] proposed an enhancement deep feature fusion method for fault diagnosis of rotating machinery. This method can fuse the features of different layers from images by neural network to further improve the quality of learning features. Gómez-Ríos et al. [45] built a classifier which can use two kinds of images, namely, texture image and structure image, to identify the species of corals. The method first identifies whether the input image is texture image or structure image by a ResNet model, and then constructs a ResNet model for texture image and structure image, respectively, to identify coral species. Wu and Li [46] proposed an automatic architecture for detecting various kidney abnormalities, in which a multifeature fusion neural network (MF-Net) was used to extract distinctive features for multiple views of images based on two input images.

All these studies have proved the importance of information fusion, but there has never been a study on the fusion of image data and structured numerical data. In this paper, a novel network structure model, FGT-Net, is proposed to improve the recognition rate of classification tasks by combining numerical data with image data.

## 3. Proposed Approach

**Figure 2:**
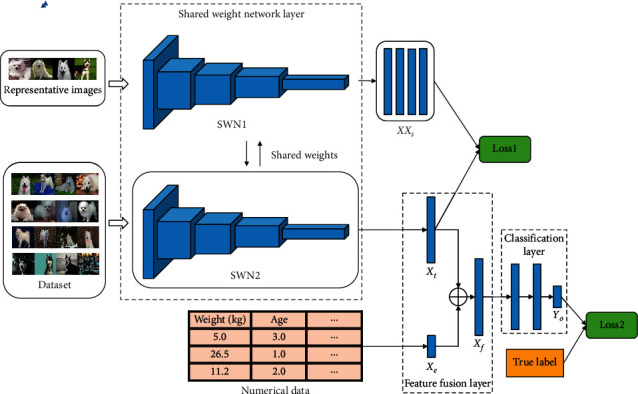
The framework of the FGT-Net model.

The framework of FGT-Net is proposed and constructed to achieve this combined function. The structure of the FGT-Net model is shown in Figure 2. It has three layers: shared weight network (SWN) layer, feature fusion layer, and classification layer. The function of shared weight network layer is to extract the feature vector of the image. Feature fusion layer is used to fuse the extracted image features and the numerical data features (features beyond the image) of the target to enhance the representativeness of the target features. After feature fusion, classification layer is used to classify the fused features and output the classification results. The training method of FGT-Net model is new: guided training. Moreover, the structures of models applied for training and testing are slightly different. The detailed processes are described as follows. The structure of the FGT-Net is introduced in Section 3.1, Section 3.2, and Section 3.3. The introduction of guided training and test is in Section 3.4.

### 3.1. Shared Weight Network Layer

As shown in Figure 2, Shared weight network layer consists of two identical CNNs, which are represented as SWN1 and SWN2, respectively, and they share weights. In the training, the input of SWN1 is representative image set *XX*_input_, and the input of SWN2 is the picture in the training set *X*_input_. In the test, only SWN2 is used to extract the image features. Representative image set refers to the image set composed of one image of each class. If there are C classes, the representative image set contains C images. Therefore, SWN1 outputs the feature vector set *XX*_*s*_=(*X*_*s*1_, *X*_*s*2_,…, *X*_*sC*_) of the representative image set, and SWN2 outputs the feature vector *X*_*t*_ of the training image. Here, *X*_*t*_, *X*_*s*1_, *X*_*s*2_, and so on are all *n*-dimensional vectors, for example, *X*_*t*_=(*x*_*t*1_, *x*_*t*2_,…, *x*_*tn*_),  *X*_*s*1_=(*x*_*s*11_, *x*_*s*12_,…, *x*_*s*1*n*_). The purpose of designing such a shared weight network layer is to make the network learn the features of each image class more directionally, that is, to learn the characteristics of specific categories of images from the representative image set, so that the features of the same image class are closer. In order to achieve our goal, a distance loss function like in [47] the following equation is designed in the output part of shared weight network layer:

(1) $$\begin{array}{c} \text{Loss}1=\text{loss}\left(X_{sc},X_{t}\right)=\frac{1}{n}\sum_{i=1}^{n}{\left(x_{sci}-x_{ti}\right)}^{2}. \end{array}$$

Here, *X*_*t*_=(*x*_*t*1_, *x*_*t*2_,…, *x*_*tn*_) represents the output of SWN2, *XX*_*s*_=(*X*_*s*1_, *X*_*s*2_,…, *X*_*sC*_) represents the output of SWN1, *c* represents the real class of input image *X*_input_, *C* represents the total number of classes, and *n* represents the dimensions of *X*_*sc*_ and *X*_*t*_.

### 3.2. Feature Fusion Layer

Feature fusion refers to the fusion of feature vectors of training images extracted from shared weight network layer and feature vectors composed of other numerical data, so that the proposed model can utilize features as many as possible for the further classification.

The features from numerical data and the features extracted by image processing techniques are both numerical values. The feature vector extracted from the image is *X*_*t*_=(*x*_*t*1_, *x*_*t*2_,…, *x*_*tn*_) ∈ *R*^*n*^; *R*^*n*^ represents an *n*-dimensional vector. As shown in Section 2.1, *X*_*t*_ is the output of SWN2. It is the feature extracted by shared weight network layer and expressed as a vector. Suppose the features obtained from numerical data are denoted as *X*_*e*_=(*x*_*e*1_, *x*_*e*2_,…, *x*_*em*_) ∈ *R*^*m*^; *R*^*m*^ represents an *m*-dimensional vector. The feature fusion is realized by the concatenation of *X*_*e*_ and *X*_*t*_, and result is represented by *X*_*f*_ that is an (*m*+*n*)-dimensional vector. The feature fusion is realized by the following formula:

(2) $$\begin{array}{c} X_{f}=X_{t}⊕X_{e}=\left(x_{t1},x_{t2},\ldots ,x_{tn},x_{e1},x_{e2},\ldots ,x_{em}\right),\;X_{f}\in R^{n+m}, \end{array}$$

where the elements (*x*_*t*1_, *x*_*t*2_,…, *x*_*tn*_ ) of *X*_*t*_ and the elements (*x*_*e*1_, *x*_*e*2_,…, *x*_*em*_) of *X*_*e*_ construct a new vector (*x*_*t*1_, *x*_*t*2_,…, *x*_*tn*_, *x*_*e*1_, *x*_*e*2_,…, *x*_*em*_) to express the fused feature vector *X*_*f*_.

### 3.3. Classification Layer

After the combination of the above image mapped features and numerical features, we can complete our classification task based on the fused features. We use several fully connected layers to achieve classification. Each neuron in the fully connected layer is fully connected with all the neurons in the previous layer. In order to improve network performance, the activation function of each neuron in the fully connected layer generally uses the ReLU function [48]. The last fully connected layer is the output layer, usually using the Softmax function as the activation function. The output layer implements the final classification. The input of classification layer is *X*_*f*_ and the output is *Y*_*O*_=(*y*_*O*1_, *y*_*O*2_,…, *y*_*OC*_), a C-dimensional feature vector in which the dimension is the same as the total number of classes. In order to make the model have better classification ability, cross-entropy loss function like in [49] is used in this paper:

(3) $$\begin{array}{c} \text{Loss}2=\text{loss}\left(Y_{O},Y\right)=-\sum_{i=1}^{C}y_{i}\log\left(y_{Oi}\right). \end{array}$$

Here, *Y*=(*y*_1_, *y*_2_,…, *y*_*C*_) represents the label of *X*_input_, where *y*_*n*_=1 if the class of *X*_input_ is *n*; for the rest, *y*=0. Loss1 can make the features of the same kind of images output by the model closer to each other, while Loss2 is the cross-entropy loss used by general classification models. In order to make the model have better classification ability, we set the loss function to guide the model training as the sum of distance loss and classification loss, which is represented by Loss_total_:

(4) $$\begin{array}{c} {\text{Loss}}_{\text{total}}=\text{Loss}1+\text{Loss}2. \end{array}$$

### 3.4. Guided Training and Test

Inspired by the method of guided filtering in [50], we adopt an unconventional training method: guided training, which is more conducive to model learning. Firstly, an image from each class in the dataset is selected as the representative image set. The remaining images are divided into training set and test set according to a certain proportion. Firstly, the representative image set is input into SWN1 to obtain the corresponding feature vector group *XX*_*s*_=(*X*_*s*1_, *X*_*s*2_,…, *X*_*sC*_); the image in the training set is input into SWN2 to obtain the feature vector *X*_*t*_=(*x*_*t*1_, *x*_*t*2_,…, *x*_*tn*_). So far, the model can calculate the Loss1. In this way, the feature vectors generated by shared weight network layer can be guided to be closer to the feature vectors of the same class in the representative image set, and let feature vectors generated by the images belonging to the same class in the model closer. This is the main purpose of our proposed guided training. Then, in order to solve the problem that only using images cannot correctly identify specific tasks (such as medical diagnosis), we propose feature fusion. The fusion feature vector *X*_*f*_ is obtained by fusing the feature vector *X*_*t*_ obtained from SWN2 and the feature vector *X*_*e*_ composed of additional numerical data. Finally, the classification layer is used to classify the fused feature vector *X*_*f*_ to get the classification result: *Y*_*O*_. In this paper, the cross-entropy loss Loss2 is used to calculate the classification loss, which makes the model learn better classification ability.

**Figure 3:**
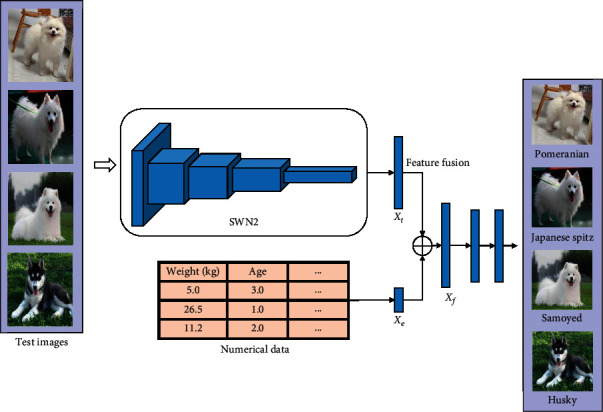
The FGT-Net model used in test.

In the test, SWN1 is no longer used shown in Figure 3, because the function of the SWN1 is to guide the model to learn the ability to obtain the image features during the training process, so that the characteristics of the images belonging to the same category are closer. Once the training is finished, the model has such ability, so this network layer is not needed in the test. When testing, we only need to put the image into SWN2, and then we can get the classification result of the image through one forward propagation.

## 4. Experiments

In order to verify the performance of the FGT-Net model method, experiments were conducted and the results of these experiments are shown below. The results of these experiments show that the FGT-Net framework can solve the classification objects. The accuracy of our FGT-Net (fused with image data and structured data) is higher than CNNs (with only image data).

### 4.1. Dataset

The images used in the experiment are collected from the Internet, the numerical data used are artificially generated according to the actual situation of each dog, and a dataset was made as shown at the end of this paper. There are four classes of data in the dataset, including class 0 (Japanese Spitz), class 1 (Pomeranian), class 2 (Samoyed), and class 3 (Husky). Each data in the dataset contains an image and 3 structured numerical data: sex, weight, and age. Sex is represented by 0 and 1 (0 for male and 1 for female). The unit of weight is kg. Samoyed's weight is less than 30 kg, Japanese Spitz's weight is below 10 kg, Pomeranian's weight is below 3.5 kg, and Husky's weight is below 30 kg. Age is based on months. If the age is less than 15 days, it is calculated as half a month, that is, 0.5 months. If it is more than 15 days, it is calculated as one month.

**Figure 4:**
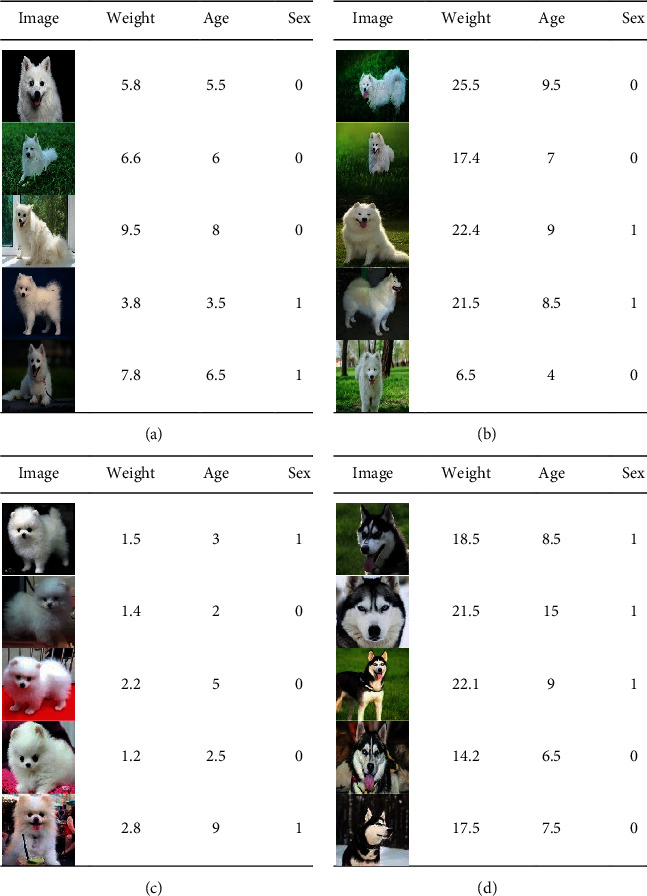
Data examples in the dataset. (a) Japanese Spitz data example (class 0), (b) Pomeranian data example (class 1), (c) Samoyed data example (class 2), and (d) Husky data example (class 3).

Among the dataset, representative image set includes 1 image of Japanese Spitz, 1 image of Pomeranian, 1 image of Samoyed and 1 image of Husky, training set including 186 images of Japanese Spitz, 107 images of Pomeranian, 330 images of Samoyed, and 353 images of Husky, and test set includes 46 images of Japanese Spitz, 27 images of Pomeranian, 84 images of Samoyed, and 89 images of Husky. Some examples of the data in the dataset are shown in Figure 4.

### 4.2. The Model

The model used in the experiment is described in this section.

First, for shared weight network layer, SWN1 and SWN2 are built for features extraction based on ShuffleNetv2. Only the front of the full connection layer of ShuffleNetv2 is used, that is, only the portion from the input layer to the average pooling layer. After the mapped features extraction, each input image can be transformed into a 1024-dimensional feature vector, which represents the mapped image features. At the same time, other numerical features from structured data corresponding to each image in the dataset are also constituted into a numerical feature vector. There are 3 numerical features in this experiment, so the numerical feature vector is a 3-dimensional feature vector. Second, the above 1024-dimensional image feature vector and 3-dimensional numerical feature vector are converted into a 1027-dimensional feature vector using the feature fusion method described in Section 2.2; then, the feature fusion is completed. Finally, two full connection layers and an output layer are added to the model, with 512 neurons, 256 neurons, and 4 neurons (corresponding to the output of 4 classes), respectively. The combined 1027-dimensional feature vector is used to complete the recognition of the object in the input image.

**Figure 5:**
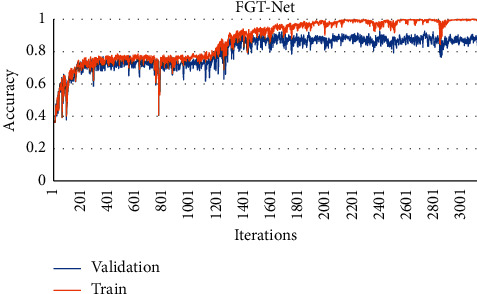
Training and verification accuracy with the number of iterations.

**Figure 6:**
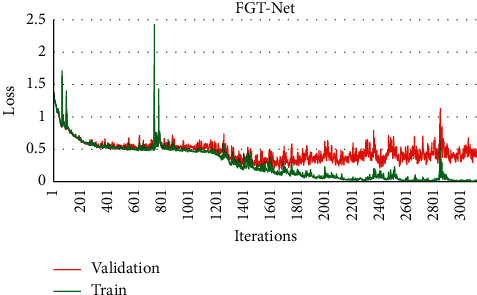
Training and verification loss with the number of iterations.

The whole training process is carried out on GPU. The loss function described in Sections 2.1 and 2.3 was used in the training of the model. Adam [51] was used as the optimizer; the initial learning rate is 0.001. There were 32 samples in each training batch of the model, 100 epochs were trained for the whole training set, and the model parameters were updated 3100 times. The training and validation accuracy figures are shown in Figure 5, and the training and validation loss figures are shown in Figure 6. As shown in Figures 5 and 6, the model converges gradually, and the change trend of the two curves is basically the same, which shows that the model can learn the characteristics of particles from the training dataset and can accurately identify the unknown wear particle samples in the verification dataset. Finally, the performance of the model is evaluated on the test set.

### 4.3. Guided Training

The purpose of this part of the experiment is to prove that the guided training is more conducive to the final classification of the model. We designed two experiments to compare, Experiment 1: only using SWN2 and classification layer, using training set training model, test set testing; Experiment 2: using SWN1, SWN2, and classification layer. The representative image set and training set are used to train the model, and the test set is used to test the model. Experiment 2 uses the guided training method to train. The only difference between Experiment 1 and Experiment 2 is whether to use SWN1 and representative image set. The experimental results are shown in Table 2. It shows that the classification accuracy of the model with guided training method (Experiment 2) is higher than that of the model learned by ordinary self-training mode (Experiment 1). It is proved that the training mode designed is more beneficial to the model training.

### 4.4. Discussion of Feature Fusion

In order to compare the performance of feature fusion, the comparison experiments were conducted with the same hyperparameter setting. In the first comparison experiment, only image data was used to train CNN model (here ShuffleNetv2 is utilized) to predict the classes of dogs. In the second comparison experiment, only structured numerical data was used to train a simple 5-layer-deep fully connected neural network (DNN) to predict the classes of dogs. The final experimental results are shown in Table 3. As can be seen from the experimental results, CNN model with only image data has relatively low recognition accuracy for Pomeranians and Japanese Spitzes when only image data was used to identify the classes of dogs. This is because the appearance, texture, color, and other characteristics of Japanese Spitzes and Pomeranians are very similar, so only using these characteristics extracted from CNN model cannot accurately identify them. Moreover, when only structured numerical data was used to identify the classes of dogs, DNN model tends to confuse Huskies and Samoyeds, and the identification accuracy of Huskies and Samoyeds is relatively low. This is because these two kinds of dogs are very similar in weight features; they cannot be well identified only by these structured numerical features. However, when the FGT-Net model was used to combine image data and structured numerical data, the two problems can be solved well. Firstly, although Pomeranians and Japanese Spitzes are similar in appearance, texture, color, and other characteristics, their weight features are quite different (Pomeranian is small sized dog and relatively light in weight; Japanese Spitz is medium sized dog and relatively heavy), so they can be identified by using structured numerical data. Secondly, although Huskies and Samoyeds are similar in weight, their appearance, texture, color, or other features are quite different, so they can be identified by using image data features. Therefore, the FGT-Net model can use both image data and structured numerical data to identify the classes of dogs. As shown in Table 3, the recognition accuracy of the FGT-Net model reaches 87.8%, the recognition accuracy of CNN (ShuffleNetv2) model which is learned only by image data is 72.8%, and the recognition accuracy of the DNN model which is learned only by structured numerical data is 78.0%. From these experimental results, we can see that the FGT-Net model can identify not only Pomeranians and Japanese Spitzes well, but also Huskies and Samoyeds well.

Because ShuffleNetV2 belongs to lightweight network, the network level is not deep. In order to further prove the effectiveness of our method, we compare FGT-Net with other well-known CNNs (ResNet50, VGG16, and AlexNet). As shown in Table 4, FGT-Net achieves the highest accuracy, because FGT-Net is based on the fused features of image data and structured data. The other CNN models (ResNet50, VGG16, and AlexNet) are produced with only image data. It proves that our method is effective in solving the problems with comprehensive data merged by image and structured data. Our method is a framework based on CNN structure, so it enlarges the function of CNN models. In terms of test time, as shown in Table 5, FGT-Net is faster than other models except that it is 0.08 seconds slower than ShuffleNetV2. Therefore, FGT-Net not only improves the accuracy, but also improves the speed.

## 5. Conclusion

In some recognition tasks, there is a problem that images could not be the only criteria for classification, or objects from different images cannot be well classified by CNN due to the similar features (color, texture, appearance, etc.). In order to solve these kinds of problems, a novel FGT-Net framework that can combine image data and structured numerical data is proposed. A guided training is adopted for the learning process so that the feature vectors generated by the similar targets are closer to each other. Therefore, FGT-Net could surpass the ordinary training method and obtain higher recognition accuracy. Experiments are executed for the fusion of image level features and numerical features extracted from structured data. The accuracy of the model with guided tra[[parms resize(1),pos(50,50),size(200,200),bgcol(156)]]et is 2.1% higher than that of the model without guided training. The accuracy of FGT-Net reaches 87.8%, which is 15% higher than CNN model of ShuffleNetv2 (which can process image data only) and 9.8% higher than DNN method (which processes structured data only). The proposed model is feasible for the future applications in the fields of industry or medical diagnosis which are considered by the merging of image data and structured data together. And the framework of the proposed model can extend the processing ability of CNNs for the merging of image data and structured data.

## Figures and Tables

**Table 1:** Acronyms and full names in this paper.

Acronyms	Full names
FGT-Net	Feature fusion network with guided training
CNN	Convolutional neural network
LRN	Local response normalization
DDE	Dynamic differential entropy
AHNF	Attribute heterogeneous network fusion
DFF-ADML	Deep feature fusion method based on adaptive discriminant metric learning
GAN	Generative adversarial network
MF-Net	Multifeature fusion neural network
SWN	Shared weight network

**Table 2:** The accuracy and other performance evaluation indexes of Experiment 1 and Experiment 2 of guided training.

Experiment	Class	TP	FP	FN	Precision	Recall	F1-score	Accuracy
Experiment 1	Japanese Spitz	10	9	36	0.526	0.217	0.308	0.707
	Pomeranian	9	4	18	0.692	0.333	0.450	
	Samoyed	69	49	15	0.585	0.821	0.683	
	Husky	86	10	3	0.896	0.966	0.930	

Experiment 2	Japanese Spitz	18	9	28	0.667	0.391	0.493	0.728
	Pomeranian	15	8	12	0.652	0.556	0.600	
	Samoyed	63	38	21	0.623	0.750	0.681	
	Husky	83	12	6	0.873	0.933	0.902	

**Table 3:** The accuracy and other performance evaluation indexes of models with fused data and separate data.

Model	Class	TP	FP	FN	Precision	Recall	F1-score	Accuracy
FGT-Net (fused data)	Japanese Spitz	40	6	6	0.870	0.870	0.870	0.878
	Pomeranian	27	6	0	0.818	1.000	0.900	
	Samoyed	71	9	13	0.888	0.845	0.866	
	Husky	78	9	11	0.897	0.876	0.886	

CNN (ShuffleNetv2) (only image data)	Japanese Spitz	18	9	28	0.667	0.391	0.493	0.728
	Pomeranian	15	8	12	0.652	0.556	0.600	
	Samoyed	63	38	21	0.623	0.750	0.681	
	Husky	83	12	6	0.873	0.933	0.902	

DNN (only structured data)	Japanese Spitz	45	0	1	1.000	0.978	0.989	0.780
	Pomeranian	27	1	0	0.964	1.000	0.982	
	Samoyed	46	15	38	0.754	0.548	0.634	
	Husky	74	38	15	0.661	0.831	0.736	

**Table 4:** Accuracy comparison with other advanced models.

Model	FGT-Net	AlexNet	VGG16	ResNet50
Accuracy	**0.878**	0.728	0.691	0.675

**Table 5:** Time comparison with other advanced models.

Model (s)	FGT-Net	AlexNet	ShuffleNetV2	VGG16	ResNet50
Time	10.39	10.57	**10.31**	11.66	10.91

## Data Availability

The data used in the experiment were collected from public on the Internet and we have built a dataset. The dataset is available at https://github.com/Fan-Suli/Datasets.
